# Beneficial effect of fluid warming in elderly patients with bladder cancer undergoing Da Vinci robotic-assisted laparoscopic radical cystectomy

**DOI:** 10.6061/clinics/2020/e1639

**Published:** 2020-04-13

**Authors:** Jianwei Luo, Lin Zhou, Shaoman Lin, Wenchan Yan, Lijuan Huang, Sihua Liang

**Affiliations:** I Department of Anesthesiology, Sun Yat-sen Memorial Hospital, Sun Yat-sen University, Guangzhou 510120, China; II Department of Intensive Care Unit, Sun Yat-sen Memorial Hospital, Sun Yat-sen University, Guangzhou 510120, China; III Department of Emergency, Sun Yat-sen Memorial Hospital, Sun Yat-sen University, Guangzhou 510120, China; IV Operating Room, Sun Yat-sen Memorial Hospital, Sun Yat-sen University, Guangzhou 510120, China

**Keywords:** Bladder Cancer, Radical Cystectomy, Hypothermia, Robotic-Assisted Laparoscopic Surgery, Fluid Warming

## Abstract

**OBJECTIVES:**

The enhanced recovery after surgery (ERAS) protocol recommends prevention of intraoperative hypothermia. However, the beneficial effect of maintaining normothermia after radical cystectomy has not been evaluated. This study aimed to investigate the efficacy of fluid warming nursing in elderly patients undergoing Da Vinci robotic-assisted laparoscopic radical cystectomy.

**METHODS:**

A total of 108 patients with bladder cancer scheduled to undergo DaVinci robotic-assisted laparoscopic radical cystectomy were recruited and randomly divided into the control group (n=55), which received a warming blanket (43°C) during the intraoperative period and the warming group (n=53), in which all intraoperative fluids were administered via a fluid warmer (41°C). The surgical data, body temperature, coagulation function indexes, and postoperative complications were compared between the two groups.

**RESULTS:**

Compared to the control group, the warming group had significantly less intraoperative transfusion (*p*=0.028) and shorter hospitalization days (*p*<0.05). During the entire intraoperative period (from 1 to 6h), body temperature was significantly higher in the warming group than in the control group. There were significant differences in preoperative fibrinogen level, white blood cell count, total bilirubin level, intraoperative lactose level, postoperative thrombin time (TT), and platelet count between the control and warming groups. Multivariate linear regression analysis demonstrated that TT was the only significant factor, suggesting that the warming group had a lower TT than the control group.

**CONCLUSION:**

Fluid warming nursing can effectively reduce transfusion requirement and hospitalization days, maintain intraoperative normothermia, and promote postoperative coagulation function in elderly patients undergoing Da Vinci robotic-assisted laparoscopic radical cystectomy.

## INTRODUCTION

Bladder cancer is the 9^th^ most common malignancy and the 13^th^ leading cause of death worldwide 1. It is primarily a disease of the elderly, with a median age of 72 years at diagnosis 2. Radical cystectomy is the gold standard treatment for muscle-invasive or high-grade non-muscle-invasive bladder cancer 3. With the development of the technique of minimally invasive surgery, Da Vinci robot-assisted radical cystectomy has become the primary and gold standard treatment for bladder cancer as it can better preserve nerve and vessels, which has been shown to reduce blood loss and transfusion, narcotic requirement, time to regular diet, and hospitalization days 3–5. However, the use of robotic-assisted surgery usually increases the overall operating time and requires prolonged artificial pneumoperitoneum and some special patient positioning, which could affect the patient's pathophysiological conditions and easily induce perioperative hypothermia, especially in the elderly 6. Intraoperative hypothermia can induce a series of perioperative complications, such as postoperative delayed wakefulness, shivering, coagulopathy, agitation, hypoxemia, poor wound healing, and headache 7. It has been shown that intraoperative hypothermia is significantly associated with a higher short-term readmission rate and mortality in elderly patients 8. Despite this, few studies exist on temperature management in Da Vinci robotic-assisted surgery.

Since infusing cold saline or packed red blood cells causes a marked decrease in patient's body temperature 9, pre-warmed infusions (to 38–42°C) is an important strategy to maintain intraoperative normothermia 10,11. The common methods of pre-warmed infusions include a double-wall infusion line and a fluid warming device 12,13. Intravenous fluids warming is one of the most effective strategies for intraoperatively maintaining normothermia 14,15. Intravenous fluid warming has been shown to effectively maintain the core temperature in laparoscopic colorectal surgery 16. The Enhanced Recovery After Surgery (ERAS) Society guidelines for perioperative care after radical cystectomy for bladder cancer strongly recommend prevention of intraoperative hypothermia 17. However, the beneficial effect of maintaining normothermia during radical cystectomy has not been evaluated^18^. Therefore, this study aimed to investigate the efficacy of fluid warming in elderly patients with bladder cancer undergoing Da Vinci robotic-assisted laparoscopic radical cystectomy.

## METHODS

### Participants and study design

A total of 108 patients with bladder cancer scheduled to undergo DaVinci robotic-assisted laparoscopic radical cystectomy were recruited into this trial. Other inclusion criteria were American Society of Anesthesiologists (ASA) Score I-III and age 50-79 years. Patients with a history of cerebral infarction, mental abnormalities, preoperative urinary tract infection, or severe lung infection or disease were excluded. This study was approved by the institutional review board of our hospital and written informed consent was waived by the Institutional Review Board due to the retrospective nature of this study.

### Fluid warming protocol

Prior to the study, all nursing staff received training for the procedure of fluid warming and the data collection. The 108 enrolled patients were randomly divided into the control group (n=55) and the warming group (n=53). All patients underwent Da Vinci robot-assisted laparoscopic radical cystectomy at room temperature under the same anesthesia treatment.

In the warmed fluid group, all intraoperative fluids were administered via the Ranger™ Blood/Fluid Warming Unit (3M, St. Paul, MN, USA) using a setpoint of 41°C. The patients in the control group used a warming blanket (setpoint=43°C) during the intraoperative period.

### Data collection

Demographic (sex, age) and baseline clinical characteristics (ASA score, Cardiac function (assessed by determining the ejection fraction), and body mass index [BMI]) were collected. Surgical information included intraoperative transfusion, intraoperative blood loss, intraoperative warming time of transfusion, operative time, anesthesia time, recovery room to extubation time, post-anesthesia care unit (PACU) stay time, postoperative flatulence, loss of blood, reoperation due to bleeding, hospitalization days, and medical expenses.

Patient's nasopharyngeal temperature was measured using the Carefusion Temperature Probe (400 Series, GE Healthcare, USA) with the Carescape Monitor B650, GE Healthcare) during the whole perioperative period. Biochemical examination results including coagulation function indexes (prothrombin time activated partial thromboplastin time, thrombin time), fibrinogen; white blood cell, platelet, total bilirubin, pH, glucose, lactose were collected during the perioperative period.

Postoperative complications including cold shivering, pneumonia, pulmonary embolism, urinary fistula, and intestinal obstruction were recorded. All data was compared between the two groups.

### Statistical analysis

Continuous variables were presented as mean±SD (standard deviation) while categorical data were indicated by number and percentage (%). Student's independent t-test was used to compare the differences between groups. If normality of continuous variables was not assumed, median (range) and non-parametric analysis Mann-Whitney U test would be used instead. Chi-square test and Fisher's exact test (if any expected value lower than 5 was observed) were used for categorical data. Two-way mixed design ANOVA was used to test the significance of main effect (time, group) and interaction effect (time*group). Univariate and multivariate linear regression models were used to investigate the significance of group factor to the differences between pre/peri-operative and postoperative index. A *p*-value lower than 0.05 would be recognized as reaching significance of each test. All analyses were performed using IBM SPSS Version 20 (SPSS Statistics V20, IBM Corporation, Somers, New York).

## RESULTS

### Demographic and clinical characteristics

A total of 108 patients (mean age= 62.20±11.05 years, range: 26-87, 82.41% male) undergoing DaVinci assisted laparoscopic radical cystectomy were enrolled, and randomly divided into the warming group (n=53) and control group (n=55). There was no significant difference in demographic and clinical characteristics between the warming and control groups (all *p*>0.05, Table 1).

### Perioperative information

The perioperative information was compared between the two groups. As shown in Table 2, the warming group had significantly less intraoperative transfusion (*p*=0.028) and shorter hospitalization days (both postoperative and total hospitalization, *p*<0.05) than the control group. However, there was no significant difference in perioperative blood loss, operative time, anesthesia time, time from entering recovery room to extubation, PACU stay time, time to postoperative flatulence, blood loss, reoperation due to bleeding, and medical expenses (all *p*>0.05).

### Change of body temperature during the perioperative period

Patient's body temperature was recorded at preoperative, intraoperative and postoperative periods. As shown in Table 3, during the entire intraoperative period (from 1 to 6h), the warming group had significantly higher body temperature than the control group (all *p*<0.05). No significance was found in preoperative and recovery room periods (*p*>0.05). At day 3 after the operation, the body temperature was slightly but significantly higher in control group than in the warming group (*p*<0.05).

### Perioperative laboratory results

Laboratory results were compared between groups. There were significant differences in preoperative FIB, white blood cell, total bilirubin; intraoperative lactose; and postoperative thrombin time (TT) between the control and warming groups (all *p*<0.05, Table 4). The difference between pre- and postoperative TT was also different between groups (*p*=0.004, Table 4).

Linear regression was used to investigate the effect of group factor on the differences between preoperative/intraoperative and postoperative results. The multivariate model was controlled with patient's age, gender, and ASA classification. The result showed that TT was the only significant factor (Table 5), suggesting that the warming group had lower TT level than the control group.

### Postoperative complications

There was no significant difference in postoperative complications between the two groups, including cold shivering in the recovery room, pneumonia, pulmonary embolism, urinary fistula, and intestinal obstruction (Table 6, all *p*>0.05).

## DISCUSSION

Under normal physiological conditions, the body core temperature fluctuates only within a narrow range (about ±0.2°C), while under anesthesia, the range is significantly increased to ±4°C 19. In addition, body temperature under anesthesia is more easily affected by external factors. Robot-assisted laparoscopic radical cystectomy increases the overall operating time and requires prolonged anesthesia 6. Most of the patients receiving radical cystectomy are elderly and are therefore more likely to develop perioperative hypothermia than young patients due to the degeneration of their body homeostasis ability. Therefore, maintaining normothermia is an important measure to reduce perioperative complications.

At present, intraoperative temperature maintaining devices include forced-air warming blankets, heating mattresses, circulating-water garment systems 20, and fluid warming system 16. The disadvantage of warming blankets and heating mattresses is that the temperature sensor of elderly patients may degenerate, which may cause burns or thermal hypersensitivity. Water in circulating-water garment systems is considered as a potential source of pathogen contamination in hospitals and can be a source of infection if not properly maintained. The fluid warming system supplies heat by inserting the infusion catheter into the metal heating plate without circulating water. Constant-temperature fluids or blood can be delivered into every part of the body. Choi et al. have demonstrated that intravenous fluid warming can effectively maintain intraoperative normothermia in patients undergoing laparoscopic colorectal surgery 16. However, the effectiveness of warming intravenous fluid has not been evaluated in robotic-assisted laparoscopic surgery.

In this study, we investigated the efficacy of fluid warming in elderly patients undergoing Da Vinci robotic-assisted laparoscopic radical cystectomy. The results showed that during the entire intraoperative period, body temperature was significantly higher in the warming group than in the control group. Especially at 6h after surgery start, the core temperature dropped to 34.9°C in the control group but still maintained at 36.01°C in the warming group. This data suggested that our fluid warming protocol can effectively maintain normothermia during operation, which is consistent with the results of the studies conducted in laparoscopic colorectal surgery 16.

In the current study, the warming group had significantly less intraoperative transfusion than the control group, suggesting that fluid warming can significantly reduce intraoperative transfusion requirement. This finding is in line with the results of a meta-analysis, including 10 studies by Rajagopalan et al., which shows that mild hypothermia (decrease by <1°C) significantly increases blood loss by approximately 16% and increases transfusion risk by 22% 21. By contrast, maintaining intraoperative normothermia can effectively reduce blood loss and transfusion requirement 21. However, although the intraoperative blood loss was less in the warming group than in the control group (141.13±108.82 *vs*. 212.73±525.31 mL) in our study, the difference did not reach statistical significance. One possible explanation might be that the advantages of reducing blood loss by fluid warming may be reduced in robotic-assisted laparoscopic surgery.

Intraoperative hypothermia has been shown to reduce the level of circulating platelets and platelets function 21,22. Supporting this notion, our study showed that the warming group had a higher postoperative platelet count compared with the control group. On the other hand, we observed that the warming group had significantly shorter postoperative TT than the control group. Multivariate linear regression analysis confirmed that the warming group had shorter TT than the control group, suggesting that fluid warming can improve the postoperative coagulation function. Intraoperative hypothermia is known to induce coagulopathy by impairing several enzymes of the coagulation cascade, eventually reducing clot formation 7. Both impaired function of platelets and coagulation can lead to increase bleeding time and intraoperative blood loss.

Yi et al. conducted a national cross-sectional study in China on 3,132 patients under general anesthesia, and the results demonstrate that intraoperative hypothermia would increase intensive care unit (ICU) admissions and prolonged hospitalization days 23. Kurz et al. have demonstrated that maintaining normothermia intraoperatively by combining intravenous fluids warming and forced-air cover can effectively decrease the incidence of infectious complications and hospitalization days 15. In agreement with these observations, our study also found that compared to the control group, the warming group had shorter hospitalization days. This observation indicated that fluid warming can promote postoperative recovery.

## CONCLUSIONS

In summary, this study demonstrated that fluid warming can effectively reduce transfusion requirement and hospitalization days, maintain intraoperative normothermia and promote postoperative coagulation function in elderly patients undergoing Da Vinci robotic-assisted laparoscopic radical cystectomy, which is worth further clinical application.

## AUTHOR CONTRIBUTIONS

The authors declare that all of them have participated actively in the study and all meet the requirements for the authorship. Liang S designed the study and wrote the protocol. Luo J, Zhou L and Lin S corrected the data. Yan W and Huang L interpreted the data. Luo J and Zhou L performed the statistical analysis. Luo J wrote the first draft of the manuscript and mainly revised the manuscript. All authors approved the final version of the manuscript.

## Figures and Tables

**Table 1:** Demographic and clinical characteristics.

Parameters	Control (n=55)	Warming (n=53)	Total (n=108)	*p*
Sex				0.240
Male	43 (78.18)	46 (86.79)	89 (82.41)	
Female	12 (21.82)	7 (13.21)	19 (17.59)	
Age, years	63.55±10.38	60.81±11.65	62.20±11.05	0.155
ASA classification				0.124
II	23 (41.82)	30 (56.60)	53 (49.07)	
III	32 (58.18)	23 (43.40)	55 (50.93)	
Cardiac function^a^	0.66±0.06	0.66±0.05	0.66±0.06	0.850
BMI	23.04±3.20	23.44±2.83	23.24±3.02	0.545

ASA, American Society of Anesthesiologists; BMI, Body Mass Index.

a Cardiac function was assessed by determining the ejection fraction (EF).

**Table 2:** Patient’s operative information.

Parameters	Control (n=55)	Warming (n=53)	Total (n=108)	*p*
Intraoperative transfusion, ml	2153.64±671.91	1903.30±491.32	2030.79±600.80	0.028
Intraoperative blood loss, ml	212.73±525.31	141.13±108.82	177.59±382.50	0.193
Intraoperative warming time of transfusion, min	-	336.43±88.91	336.43±88.91	-
Operative time, min	346.51±94.85	322.57±81.29	334.76±88.86	0.190
Anesthesia time, min	507.38±104.83	476.68±93.28	492.31±100.06	0.132
Recovery room to extubation time, min	26.39±12.70	26.62±14.31	26.50±13.44	0.909
PACU stay time, min	70.17±19.55	75.91±27.84	72.90±23.90	0.410
Postoperative flatulence, day	3.71±1.71	3.70±1.39	3.70±1.55	0.768
Loss of blood, ml				
Operative day	429.42±214.71	431.32±164.92	430.35±191.01	0.268
Day 1 after surgery	434.04±274.84	421.36±181.62	427.81±232.79	0.683
Day 2 after surgery	326.73±203.61	313.72±180.74	320.34±191.94	0.822
Day 3 after surgery	300.27±269.22	312.06±271.74	305.83±269.16	0.718
Reoperation due to bleeding				0.580
No	53 (96.36)	52 (98.11)	105 (97.22)	
Yes	2 (3.64)	1 (1.89)	3 (2.78)	
Hospitalization, days				
Postoperative	20 (5-75)	16 (5-48)	18 (5-75)	<0.001
Total	34.07±10.87	23.62±6.82	28.94±10.48	<0.001
Medical expenses				
Anesthesia	12056.21 (9285.64-23325.39)	12861.46 (7923.50-19068.38)	12478.44 (7923.50-23325.39)	0.557
Total	107026.61 (79421.88-359872.75)	103629.18 (82531.73-183466.73)	105198.58 (79421.88-359872.75)	0.823

PACU; post-anesthesia care unit.

**Table 3:** Change in the patient’s body temperature during the perioperative period.

Parameters	Control (n=55)	Warming (n=53)	Total (n=108)	*p*
Preoperative	36.45±0.36	36.48±0.35	36.47±0.35	0.641
Intraoperative				
1 hour	36.05±0.42	36.22±0.47	36.13±0.45	0.036
2 hours	35.80±0.39	36.14±0.45	35.97±0.45	<0.001
3 hours	35.54±0.44	36.10±0.40	35.81±0.50	<0.001
4 hours	35.27±0.39	36.09±0.39	35.67±0.56	<0.001
5 hours	35.06±0.34	36.12±0.30	35.55±0.62	<0.001
6 hours	34.90±0.30	36.01±0.33	35.29±0.62	<0.001
Postoperative				
In recovery room	36.25±0.38	36.12±0.38	36.19±0.38	0.103
Back to the ward	36.42±0.37	36.47±0.39	36.44±0.38	0.564
Day 1	37.07±0.44	36.95±0.43	37.01±0.44	0.160
Day 2	36.92±0.42	36.87±0.32	36.89±0.37	0.310
Day 3	36.93±0.54	36.74±0.30	36.84±0.45	0.024

**Table 4:** Laboratory results during perioperative period.

Parameters	Control (n=55)	Warming (n=53)	Total (n=108)	*p*
Preoperative				
PT	11.83±0.82	11.61±0.92	11.72±0.87	0.121
APTT	25.59±4.33	25.34±4.00	25.46±4.15	0.907
TT	18.23±2.82	17.32±1.86	17.78±2.43	0.080
FIB	3.78±2.92	3.07±1.17	3.43±2.25	0.034
WBC	6.51±2.06	7.78±2.90	7.14±2.58	0.023
Platelet	229.96±59.78	259.09±71.17	244.26±66.92	0.056
Total bilirubin	13.36±4.91	11.13±3.93	12.27±4.58	0.012
Intraoperative (average)				
PH	7.31±0.06	7.33±0.05	7.32±0.06	0.210
GUL	6.27±1.12	6.26±1.13	6.27±1.12	0.875
LAC	0.69±0.20	0.85±0.39	0.77±0.31	0.016
Postoperative				
PT	15.44±13.46	12.33±0.89	13.99±9.91	0.069
APTT	30.41±9.21	27.14±4.84	28.89±7.62	0.245
TT	17.84±5.19	15.72±1.48	16.85±4.04	0.010
FIB	3.67±1.40	3.32±0.99	3.50±1.23	0.450
WBC	11.05±4.30	12.22±4.12	11.62±4.24	0.219
Platelet	187.91±53.33	229.83±67.46	208.48±63.95	0.002
Total bilirubin	14.26±5.90	13.59±6.81	13.93±6.34	0.378
pH	7.34±0.04	7.35±0.04	7.35±0.04	0.084
GUL	7.18±1.75	7.10±1.98	7.14±1.85	0.453
LAC	1.18±0.68	1.25±0.69	1.21±0.68	0.414
Difference between preoperative and postoperative period				
PT	3.50±13.40	0.66±1.13	2.18±9.86	0.156
APTT	4.14±6.30	1.53±3.89	2.93±5.43	0.127
TT	0.26±4.44	-2.14±2.42	-0.86±3.81	0.004
Fibrinogen	0.24±1.23	0.46±1.39	0.35±1.30	0.457
WBC	4.53±4.33	4.43±3.67	4.48±4.01	0.939
Platelet	++	-29.26±36.51	-35.78±36.56	0.161
Total bilirubin	0.89±5.58	2.46±5.83	1.66±5.73	0.265
pH	0.03±0.06	0.02±0.05	0.03±0.05	0.887
Glucose	0.87±1.48	0.85±1.54	0.86±1.50	0.430
Lactose	0.48±0.69	0.42±0.65	0.46±0.67	0.958

PT, prothrombin time; APTT, activated partial thromboplastin time; TT, thrombin time; WBC, white blood cell; Platelet; pH.

**Table 5:** Univariate and multivariate linear regression results.

	Linear regression			
	Univariate		Multivariate	
	B (95% CI)	*p*	B (95% CI)	*p*
PT	-2.84 (-8.03-2.34)	0.277	-3.11 (-8.72-2.51)	0.272
APTT	-2.61 (-5.42-0.19)	0.067	-1.59 (-4.37-1.20)	0.259
TT	-2.40 (-4.32--0.48)	0.015	-2.46 (-4.47--0.44)	0.018
Fibrinogen	0.22 (-0.47-0.92)	0.522	0.30 (-0.45-1.05)	0.426
WBC	-0.10 (-1.63-1.44)	0.900	-0.08 (-1.64-1.48)	0.920
Platelet	12.79 (-1.01-26.59)	0.069	11.17 (-2.83-25.17)	0.117
Total bilirubin	1.57 (-0.61-3.74)	0.156	1.53 (-0.71-3.77)	0.178
pH	0.00 (-0.03-0.02)	0.743	0.00 (-0.03-0.02)	0.871
Glucose	-0.02 (-0.65-0.61)	0.952	0.03 (-0.63-0.68)	0.935
Lactose	-0.06 (-0.34-0.22)	0.677	0.00 (-0.29-0.28)	0.975

PT, prothrombin time; APTT, activated partial thromboplastin time; TT, thrombin time; WBC, white blood cell; Platelet; pH.

**Table 6:** Postoperative complications.

Parameters	Control (n=55)	Warming (n=53)	Total (n=108)	*p*
Cold shiver in recovery room				0.282
No	38 (70.37)	39 (79.59)	77 (74.76)	
Yes	16 (29.63)	10 (20.41)	26 (25.24)	
Pneumonia				0.963
No	52 (94.55)	50 (94.34)	102 (94.44)	
Yes	3 (5.45)	3 (5.66)	6 (5.56)	
Pulmonary embolism				-
No	55 (100.00)	53 (100.00)	108 (100.00)	
Yes	0	0	0	
Urinary fistula				0.687
No	39 (70.91)	35 (67.31)	74 (69.16)	
Yes	16 (29.09)	17 (32.69)	33 (30.84)	
Intestinal obstruction				0.378
No	37 (67.27)	39 (75.00)	76 (71.03)	
Yes	18 (32.73)	13 (25.00)	31 (28.97)	
